# Gallbladder Pneumatosis With Pneumoperitoneum Caused by Clostridium perfringens: A Case Report

**DOI:** 10.7759/cureus.93936

**Published:** 2025-10-06

**Authors:** Bethany J Ashton, Subiksha Subramonian, Manoj Nair

**Affiliations:** 1 General Surgery, North Middlesex University Hospital, London, GBR

**Keywords:** cholecystitis, clostridium perfringens, emphysematous cholecystitis, laparoscopic cholecystectomy, pneumoperitoneum

## Abstract

Emphysematous cholecystitis (EC) is a rare, life-threatening variant of acute cholecystitis characterised by gas formation within the lumen and walls of the gallbladder. This reflective case report describes a rare presentation of EC complicated by pneumoperitoneum, without free bile or identifiable perforation. It highlights the diagnostic challenges and the management considerations associated with this severe condition. A 64-year-old male with type 2 diabetes mellitus, hypertension, obesity and other comorbidities presented with a six-day history of abdominal pain and bloating. Blood tests revealed marked leukocytosis with neutrophilia and an elevated C-reactive protein. Despite an unremarkable abdominal exam, CT imaging revealed gas within the gallbladder lumen, walls and free intraperitoneal air, without associated free fluid or bile. Emergency laparoscopic cholecystectomy revealed a necrotic, gas-containing gallbladder and localised pus, but no perforation. The patient recovered well with targeted antibiotic therapy. This case highlights that pneumoperitoneum in the absence of free bile or visible perforation can occur in EC. Awareness of this atypical presentation is critical to avoid diagnostic delay and ensure timely surgical management.

## Introduction

Emphysematous cholecystitis (EC) is an uncommon, severe form of acute cholecystitis characterised by the presence of gas within the gallbladder lumen and walls, produced by gas-forming organisms such as *Clostridium* species and *Escherichia coli *[1]. It carries significantly higher morbidity and mortality compared to uncomplicated cholecystitis, with reported mortality rates ranging from 15% to 25%, and morbidity rates of up to 50% in some series [2,3]. Complications such as gangrene, perforation and sepsis are common if not promptly managed [4]. EC is most frequently observed in elderly, diabetic or immunocompromised patients and often requires urgent surgical intervention [4].

Clinically, EC may present with right upper quadrant pain, fever and leukocytosis; however, presentation can be nonspecific, particularly in patients with comorbidities such as diabetes mellitus, which may mask classical symptoms [5,6]. This contributes to the often delayed diagnosis and highlights the pivotal role that computed tomography (CT) imaging plays in diagnosis.

While EC is well known to cause significant complications, including sepsis, gangrene and gallbladder perforation, the presence of pneumoperitoneum is uncommon. When detected, pneumoperitoneum usually raises concern for gastrointestinal perforation or biliary-enteric fistula; however, its occurrence in EC is rare and often unexplained [3].

We present a 64-year-old male with EC complicated by pneumoperitoneum, in the absence of visible gallbladder perforation or bile leakage. This unusual case highlights the importance of early imaging, surgical intervention and clinical vigilance in managing this rare and potentially fatal condition.

## Case presentation

A 64-year-old male presented to the emergency department with a six-day history of progressive abdominal pain and bloating. He had attended twice in the preceding week and was initially treated for a urinary tract infection and later gastroenteritis. He re-presented with worsening symptoms despite analgesia. His medical background included insulin-dependent type 2 diabetes mellitus, hypertension, hypercholesterolaemia, a learning disability and depression. He had no prior history of gallstone disease.

On arrival, the patient was hypoxic. His observations were as follows: oxygen saturation, 93% on 3L oxygen; blood pressure, 142/78 mmHg; respiratory rate, 18 breaths/min; heart rate, 78 bpm; and he was afebrile. Abdominal examination revealed right upper quadrant tenderness and a markedly distended, tympanic abdomen. No signs of peritonitis were present, and Murphy’s sign was negative.

Initial laboratory investigations (Table 1) demonstrated leukocytosis with neutrophilia and a significantly elevated C-reactive protein. Renal and liver function tests were within normal limits. Serum lactate was at the upper limit of normal, and the glucose level was markedly elevated.

**Table 1:** Laboratory Results on Admission.

Test	Result	Reference Range
White Blood Cells	19.2 ×10^9^/L	4.0-11.0 ×10^9^/L
Neutrophils	16.2 ×10^9^/L	2.0-7.5 ×10^9^/L
CRP	228 mg/L	<5.0 mg/L
Bilirubin	10 μmol/L	0-20 μmol/L
Lactate	2.4 mmol/L	0.6-2.4 mmol/L
Glucose	21.2 mmol/L	4.0-7.8 mmol/L

**Figure 1:**
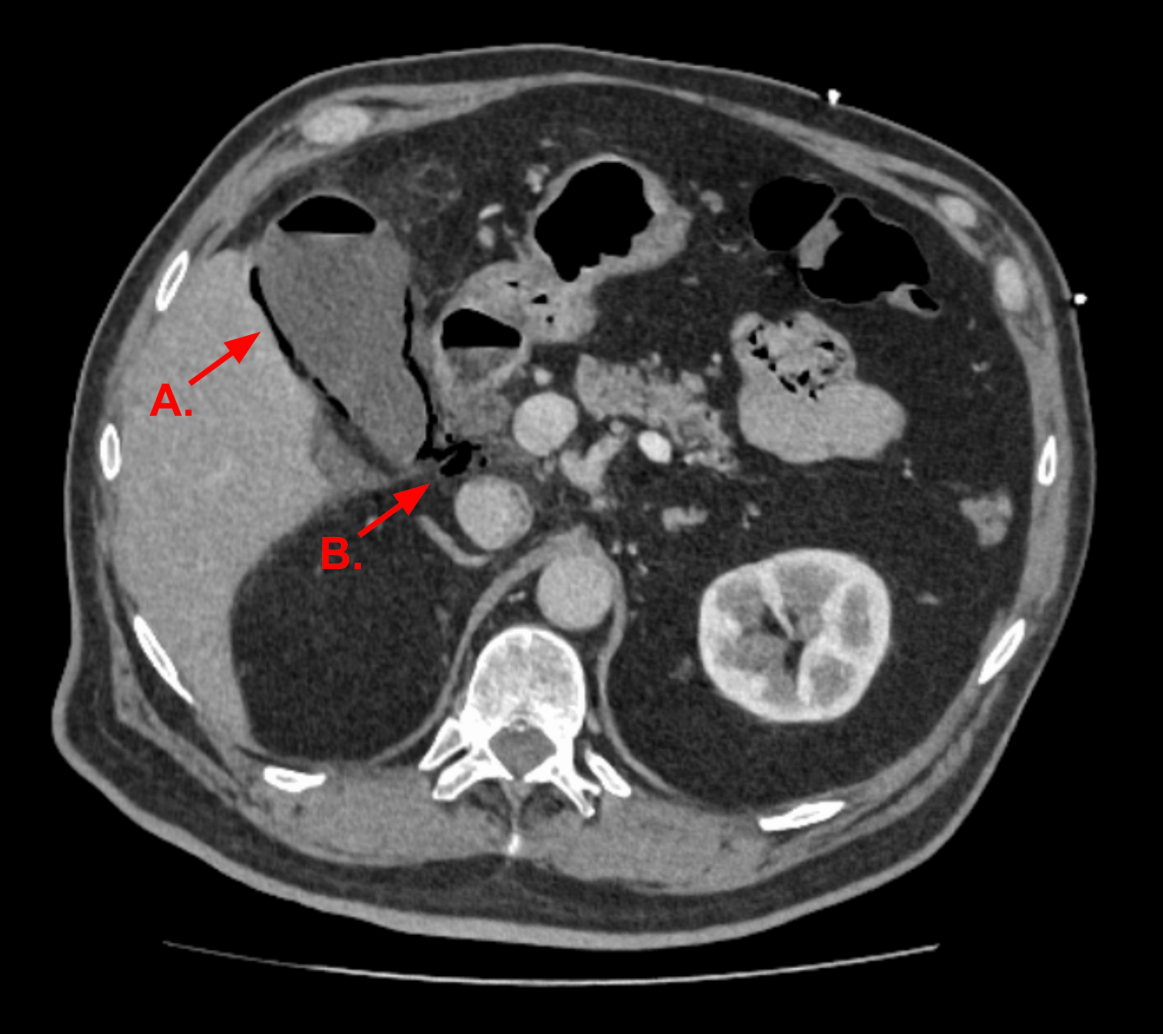
CT Abdomen and Pelvis With Contrast. The gallbladder wall demonstrated extensive gas content (A) associated with small pneumobilia. A few locules of pneumoperitoneum were also visible in the upper abdomen (B).

Given the patient’s undifferentiated abdominal pain, along with abnormal blood tests, a contrast-enhanced CT scan of the abdomen and pelvis was performed to evaluate for a broad range of potential intra-abdominal causes. This demonstrated features consistent with EC, including intramural gas (Figure 1). Pneumoperitoneum, pneumobilia and pericholecystic fluid were also noted.

**Figure 2:**
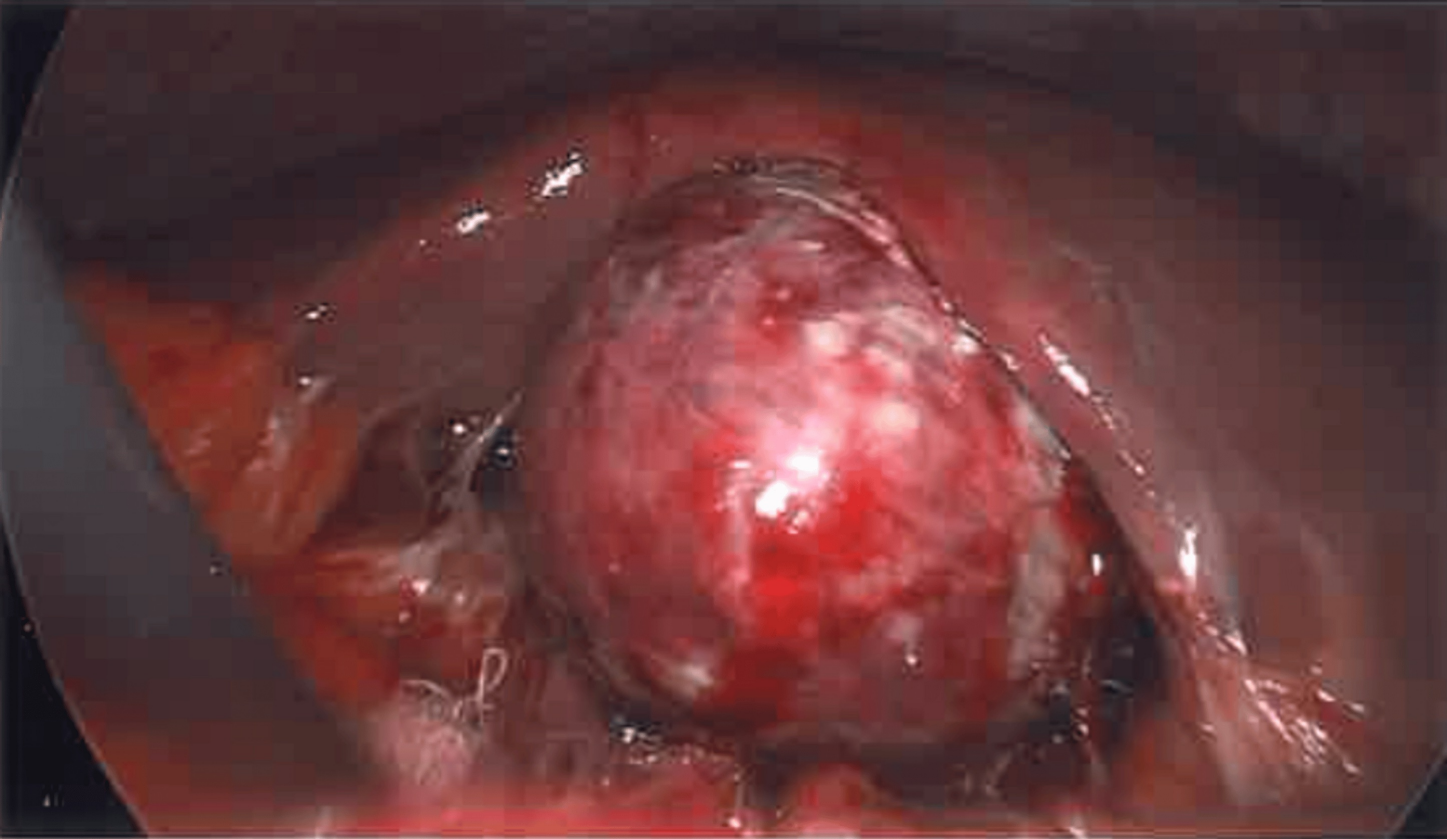
Intraoperative Image From Laparoscopic Cholecystectomy. The gallbladder appeared acutely inflamed and necrotic.

The patient was admitted and underwent an emergency laparoscopic cholecystectomy the following morning. Intraoperatively, the gallbladder appeared acutely inflamed and necrotic, with surrounding free pus, but no gallbladder perforation or fistula was visualised (Figure 2). The omentum was initially sealed around the gallbladder, and reactive fluid was seen surrounding the liver. The gallbladder was decompressed with a Veress needle, and bile was sent for microscopy, culture and sensitivity testing. The surgery was otherwise uncomplicated.

Empirical intravenous antibiotics were initiated to provide broad-spectrum cover (piperacillin-tazobactam and metronidazole). Microscopy cultures grew *Enterococcus faecalis* and *Clostridium perfringens*. Based on sensitivity results and microbiology consultation, antibiotic therapy was subsequently de-escalated to intravenous co-amoxiclav and metronidazole. The patient completed a 14-day course of antibiotics in total.

Histological examination of the resected gallbladder confirmed the diagnosis of acute gangrenous cholecystitis. The gallbladder measured 78x35x36mm and was grossly necrotic macroscopically, with a grey-tan haemorrhagic appearance. Two small gallstones were recovered from the lumen. Microscopically, there was evidence of transmural inflammation, necrosis and haemorrhage. Mild reactive atypia was noted in the biliary epithelium without dysplasia, intestinal metaplasia or malignancy.

Postoperatively, the patient's recovery was uneventful. After a 13-day hospital stay, he was discharged without complications. At follow-up four weeks later, he remained well with improved blood test results.

## Discussion

EC is a rare and life-threatening variant of acute cholecystitis, first described at autopsy by Stolz in 1901, and then radiographically by Hegner in 1931 [7,8]. It occurs in only 1% to 4% of cases of acute cholecystitis [9,10]. The current total number of cases documented in medical literature is not readily available, with most studies consisting of individual case reports and small case series, highlighting the rarity of this condition.

Unlike traditional cholecystitis, EC has a higher prevalence in males, with a male-to-female ratio of 7:3 [2]. EC occurs more frequently in patients aged 50 to 70 years, with the mean age of diagnosis being 60 years. EC carries a much higher perforation risk than acute cholecystitis [3]. Other complications of EC include gangrene and pericholecystic abscess. The mortality rate of EC is reported to be 15-25%, whereas that of uncomplicated acute cholecystitis has been reported as 1.4% [2,3]. EC is characterised by the presence of gas accumulating within the gallbladder wall or lumen, primarily due to infection by gas-forming organisms such as *E. coli, Clostridium spp., Klebsiella spp.* and other anaerobic organisms [5].

Fifty per cent of patients with EC have a history of diabetes mellitus [4]. Diabetes mellitus is also a predisposing factor to other emphysematous conditions of the abdomen, such as emphysematous pyelonephritis and emphysematous cystitis [1]. Environments with rich glucose supply encourage the growth of anaerobic bacteria, which produce gas in the presence of ischaemic tissue [5].

Compromise of the cystic artery or its branches can also predispose a person to EC. Atherosclerosis, vasculitis and/or thromboembolism are all proposed mechanisms [6]. Subsequent gallbladder ischaemia and necrosis can result in susceptibility to gas-forming bacteria proliferating, causing luminal and intramural gas collections.

In addition to diabetes mellitus and vascular compromise, various other risk factors for EC have been described. Immunosuppression of varying causes (chemotherapy, malignancy, chronic corticosteroid use, advanced HIV infection) can predispose patients to gas-forming infections [5,9]. Chronic kidney disease, most notably in those undergoing dialysis, has been associated with an increased risk of EC, likely due to impaired immune response [6]. Other epidemiological risk factors, including male sex, advancing age and metabolic syndrome, have been associated with increased risk [1,2]. Finally, delayed presentation and inadequate management of acute cholecystitis are important factors, as gallbladder ischaemia and necrosis create an environment susceptible to gas-forming organisms [3,10].

In this case, the patient’s multiple cardiovascular risk factors, including hypertension, type 2 diabetes mellitus and hypercholesterolemia, likely contributed to the development of EC. The presentation of EC can be indistinguishable from that of uncomplicated acute cholecystitis. Symptoms typically include right upper quadrant abdominal pain, fever, nausea and vomiting. Symptoms can be especially subtle in those with diabetes mellitus and end-stage renal disease [6]. Diabetic autonomic neuropathy can blunt visceral pain perception, while hyperglycaemia impairs neutrophil function, both of which may delay presentation or mask typical symptoms [6]. In our patient, classical signs such as fever and guarding were absent, possibly due to these mechanisms. This emphasised the need for heightened clinical suspicion in diabetic patients presenting with non-specific abdominal complaints.

Diagnosis hinges on imaging to identify the presence of gas within the gallbladder lumen, wall and/or biliary tract. Recent biliary tract diagnostic or therapeutic interventions need to be excluded as a potential cause of these findings. While ultrasound scans are often the first-line investigation, it has limited sensitivity in EC due to poor visualisation caused by gas artefacts [3].

CT imaging offers superior diagnostic accuracy, with a reported sensitivity of ~100% in the diagnosis of EC [4,5]. It can allow for EC to be radiologically classified into three distinct stages (Table 2). Additional CT findings suggestive of EC include pneumobilia, pericholecystic fluid, irregular gallbladder wall and hepatic abscess formation [11,12]. Our patient exhibited stage III EC, complicated by pneumoperitoneum.

**Table 2:** Stages of Emphysematous Cholecystitis.

Stage	CT Findings
Stage I	Intraluminal Gas
Stage II	Intramural Gas
Stage III	Pericholecystic Gas

Pneumoperitoneum in the setting of EC is very rare, with fewer than 25 cases reported in English-language medical literature [2]. In this case, the absence of visible perforation or bile-stained fluid raises the possibility of microperforation, transmural gas migration, or sealed perforation. Alternative causes such as gas gangrene were considered, but lack of widespread tissue necrosis and the patient’s swift recovery argue against it. One case report, which described a case of EC complicated by gas gangrene, described massive pneumoperitoneum, widespread gangrene intraoperatively and a more complicated postoperative period [2].

Emergency cholecystectomy is the definitive treatment for this condition. Traditionally, open cholecystectomy has been favoured due to inflammation that can obscure Calot’s triangle. However, laparoscopic cholecystectomy is increasingly accepted in stable patients without evidence of diffuse peritonitis or gangrene. Percutaneous cholecystostomy can serve as a bridge to definitive, second-stage cholecystectomy [3]. In high-risk surgical candidates, percutaneous cholecystostomy combined with broad-spectrum antibiotics may provide an appropriate alternative [3]. Conservative management with antibiotics, intravenous fluids, and analgesia has proven successful in some cases [13].

## Conclusions

EC is a rare but life-threatening condition requiring high clinical suspicion, especially in diabetic patients who may present atypically due to blunted inflammatory and pain responses. This case highlights an unusual case of EC complicated by pneumoperitoneum in the absence of visible gallbladder perforation or bile leakage, emphasising that such a finding does not always signify hollow viscus perforation. Prompt CT imaging and early laparoscopic cholecystectomy led to a favourable outcome. This case adds to the limited literature on atypical presentations of EC and underscores the importance of early diagnosis and surgical intervention in high-risk patients. Larger, multi-centre studies are warranted to better understand the clinical spectrum and guide management strategies for this rare but serious condition.
